# Nurses’ perception of anticipated nursing care: A qualitative research

**DOI:** 10.1371/journal.pone.0308257

**Published:** 2025-02-12

**Authors:** Khadijeh Dehghani, Imane Bagheri, Atena Dadgari, Naiire Salmani

**Affiliations:** 1 Nursing Faculty, Nursing and Midwifery School, Shahid Sadoughi University of Medical Sciences, Yazd, Iran; 2 Nursing Faculty, Meybod Nursing School, Shahid Sadoughi University of Medical Sciences, Yazd, Iran; Alexandria University Faculty of Nursing, EGYPT

## Abstract

**Background:**

Anticipated nursing care is provided significantly earlier than expected by nurses, caregivers, and other healthcare staff for patients. This type of care is influenced by many factors and is followed by various positive and negative consequences. Accordingly, the present study sought to explore nurses’ perceptions of anticipated nursing care.

**Methods:**

This qualitative study was conducted using content analysis on twelve nurses from different internal and surgical wards of Shahid Sadoughi Hospital, Yazd, Iran from November 2022 to September 2023The participants were selected using purposive sampling. The data were collected through semi-structured interviews with the nurses and analyzed using Graneheim and Lundman’s qualitative content analysis method.

**Results:**

Data analysis revealed four main categories and eleven subcategories. The main categories included *early and priority care* with two subcategories, *scope of anticipated care* with four subcategories, *predictors of anticipated care* with three subcategories, and *outcomes of anticipated care* with two subcategories.

**Conclusions:**

Based on the findings, it is suggested that nursing managers must focus on the antecedents of anticipated care and address strategies to improve the working conditions of nurses, changing routine procedures for providing care and the doctor-nurse interaction, developing and organizing training programs on clinical reasoning, decision-making and time management for nurses.

## Background

Nursing, as an integral part of the healthcare system, involves procedures to promote health, prevent diseases, and provide care for patients with physical and psychological disorders and disabilities of all ages. The effective provision of care at the front line of nursing services leads to a higher level of satisfaction and promotion of the health status of patients [1]. Besides, one of the rights of hospitalized patients is to ensure their needs are met and they receive safe and comprehensive healthcare [2] So nurses spend the most time with patients among all medical professionals and provide a wide range of services under the holistic care framework [3].

In recent years, concepts such as missed nursing care [4, 5], Unfinished nursing care [6, 7], incomplete nursing care [8, 9], rating of nursing care [10, 11], and compromised nursing care [12] have been studied by several researchers. Conceptually, missed care is defined as any aspect of the patient’s required care that has been omitted (either partially or completely) or delayed [4, 5] and is related to the lack of resources and a combination of skill imbalance [12–15], implicit and explicit rules of the team [16], nurses’ decision-making processes [11], increase in the number of patients [17], and communication issues within the care team [5]. Considering the factors leading to missed care, the lack of time to decide which care should be given priority is a determining factor [9].

As a result, some care is more likely to be delayed or omitted [16, 17]. Accordingly, some studies have focused on identifying predictors and effective interventions to reduce the occurrence of missed care [18]. However, some narrative data and evidence have shown that nurses, instead of delaying or omitting care, tend to predict some nursing interventions. Clear examples of this type of care can be found when nurses wake up patients early in the morning, provide the healthcare needed by the patients very early in the morning, or prepare the patients the night before to perform some activities before breakfast.

There is also evidence showing that nurses start actions such as preparing medications by placing them on the patient’s bedside tables and administering them ahead of time [19] or despite the recommendations that antibiotic prophylaxis is prescribed in dirty surgeries and implants should be performed within 60 minutes before incision, experimental evidence has shown that prophylaxis is performed more than 120 minutes before incision [20]. There are also reports of placing peripheral cannulas in the emergency department next to newly admitted patients to ensure venous access, while about 18.2% of them are not used later [21], showing that this intervention has been performed before the appointed time without the need for its performance [18]. Thus, such evidence highlights a phenomenon that is strongly an outcome of the nursing care prioritization process [22] in which nurses consider order, importance, or time to perform competing care and begin their work with some care, such as administering antibiotic prophylaxis, and end with those at risk of delay or omission [8, 9, 22].

And the available anecdotal and empirical examples show that the process of prioritizing nursing care can highlight the emergence of a phenomenon that is completely the result of it [22]. A review of the literature indicates that for the first time, Bogatta et al. (2020) introduced anticipated nursing care as the main finding of a qualitative study. Anticipated nursing care is a type of care provided to patients significantly earlier than expected by nurses, caregivers, and other members of the care team. Medication administration, moving patients, healthcare, dressing changes, vital parameters monitoring, blood sampling, and management activities are among the interventions that are performed before the expected time. Besides, the reasons for anticipated nursing care include individual values and attitudes, group attitudes, including always being ready for the “unexpected”, implicit group norms regarding leaving the patient and the ward after completing all the required procedures, high workload, intertwined activities, and procedures in the ward [18].

The provision of anticipated nursing care can also bring some positive outcomes, including patients’ satisfaction with nurses who perform care ahead of time and consider it a sign of attention and activeness of nurses. Moreover, reducing the occurrence of missed care can be one of the other positive outcomes of this type of care. For example, early administration of drugs reduces the possibility of delayed administration or non-administration. In addition, anticipated nursing care enables nurses to manage unpredictable problems and activities of the ward, and by promoting teamwork within the ward, creates cohesion and a sense of respect, especially when the nurses of the next shift are novices and inexperienced and are not agile in providing the care needed by the patients. Moreover, anticipated nursing care helps nurses complete some care before the medical team begins their activities and this technique is considered desirable in multidisciplinary activities [18].

However the provision of anticipated nursing care can lead to some negative consequences, including disruption of the patient’s sleep pattern, changes in the pharmacodynamics of some drugs, threatening the patient’s daily rhythms contrary to the nurses’ care goals [23], and reducing the quality of nursing care [18]. In addition, the delivery of anticipated nursing care can also affect the education of nursing students because they tend to repeat the behavior patterns learned from the nurse preceptor and it strengthens their belief that anticipated nursing care is an acceptable and effective care measure, and this attitude may be reinforced after graduation [24].

The provision of anticipated nursing care is influenced by many factors and it has some positive and negative effects. Furthermore, most nurses in Iran tend to focus on the physical dimensions of care, while nurses are required to provide comprehensive care to patients [25] and an investigation of nursing care can lead to reforms in service delivery and the provision of nursing care following standard models. Moreover, studies can focus on the tendency to improve quality in the healthcare sector, discover weaknesses and strengths of care delivery, and pave the way for the provision of higher-quality care to satisfy the patients [26].

Given that anticipated care is a context-dependent phenomenon, and knowing the context is of great importance for a better understanding of the concept, anticipated nursing care is often provided depending on the relevant context/setting and various personal and physical resources in the healthcare system. An issue of interest is what constitutes anticipated care in developing countries with limited resources and which factors influence its occurrence. To answer such questions, qualitative research methods can provide unique opportunities to understand complex and delicate situations where there is interpersonal ambiguity and multiple interpretations, create a better understanding of the phenomenon, and provide a direction for future studies [27]. The objective of the present study was to explain nurses’ perceptions of anticipated nursing care.

## Methods

### Design

This qualitative study was carried out from November 2022 to September 2023 in various internal surgical wards of Shahid Sadoughi Hospital in Yazd,Iran. The study was approved by the Ethics Committee of Shahid Sadoughi University of Medical Sciences, ethical code IR.SSU.REC.1401.065.Qualitative research uses reliable approaches to exploring and describing complex phenomena, presenting textual reports from people’s lives, and understanding concepts [28]. The present study adopted a qualitative exploratory and conventional content analysis approach. This approach systematically classifies the collected data and discovers the themes or patterns that are apparent and hidden in the data [29].

To ensure the quality of the research procedure, this study followed 32 items of the consolidated criteria for reporting qualitative research checklist.

### Setting and sampling

The participants in the study were nurses working in these wards. The criteria for enrollment in the study were having at least six months of work experience in the ward and the ability to communicate and share experiences. The participants could withdraw from the study at any time they wished. The participants were selected using purposive sampling with maximum variation in terms of demographic characteristics. The participants were selected from different age groups with varying degrees of work experience including native, non-native, single, and married nurses working under various official, contractual and plan based.

### Data collection

The data in this study were collected through interviews with 12 nurses and the interviews continued until the data were saturated. The interviews were conducted upon prior arrangements with the participants at the beginning or end of their shifts in the nursing restroom. Before conducting the interviews, some instructions were provided to the participants about the objectives of the study, the procedure taken to conduct the interviews, the recording of the participants’ statements, the probable need for conducting further interviews, the anonymity and confidentiality of the participants’ data, and voluntary participation in the study.

All procedures were performed in line with the relevant guidelines and regulations and compliance with the Declaration of Helsinki Consent for publication. The interviews began after obtaining oral and written informed consent from the participants.

Semi-structured interviews were used in the study for data collection. Examples of the questions asked in the interviews were as follows: What comes to your mind when you hear anticipated nursing care? What care do you routinely perform? Why do you predictably provide some care? What are the results of providing this type of care?

During the interviews, probing questions were also asked to elicit more information and clarify any ambiguity in the participants’ statements. At the end of the interviews, the participants were asked to add any further comments if they wished, and they were appreciated for attending the interview session. The participants were also informed that if the research would come up with new questions after transcribing the content of the interviews, they would be invited for additional interviews. Each interview lasted 45 to 90 minutes, with an average time of 55 minutes.

### Data analysis

Data analysis was performed simultaneously with data collection by MAXQDA-10 software using the conventional content analysis method (Graneheim & Lundman, 2004) [29]. After completing the interviews, the researcher listened to the audio files of the interviews several times and transcribed the content of the interview word by word on paper. Then, the text of the interview was read several times to understand and identify the overt and covert themes in it and the identified themes were coded. Afterward, the extracted codes were compared in terms of similarities and differences, and the codes with similar meanings were clustered in a single group to form subcategories. In the next step, the developed subcategories were compared, and similar subcategories were placed into a new category. This process continued until the last interview when all extracted codes and themes were placed into the relevant categories and subcategories.

### Rigor

Guba and Lincoln’s (2011) criteria (credibility, dependability, transferability, and confirmability) were used to check the rigor of the findings [30].

To enhance the credibility of the data, after conducting the interviews and initial coding, the findings of the study were reviewed by the participants and revised based on the feedback received from the participants (member checking) and to ensure the reliability of the findings, in addition to the researchers, several qualitative research experts were also asked to review the texts to evaluate the accuracy of the data analysis process (peer checking).

To ensure the dependability of the findings, an external observer reviewed the extracted codes and categories and compared them with an excerpt from the interviews. Any disagreement or inconsistency in the findings was resolved based on the feedback provided by the observer. To improve the transferability of the findings, several nurses who did not participate in the study were provided with the findings, and they compared and matched the findings with their experiences. Finally, to check the conformability of the findings, the extracted themes and categories were reviewed and confirmed by some external observers who did not participate in the study.

## Results

The age of the nurses participating in this study ranged from 23 to 50 years and all the participants held a bachelor’s degree. Characteristics of study participants described in "Table 1".

**Table 1:** Characteristics of study participants.

number	sex	Employment Status	ward	work experience
1	woman	plan based	oncology	13 months
2	woman	Official	oncology	13 years
3	woman	Official	heart	12 years
4	Man	plan based	nerves	2 years
5	Man	plan based	General medical	8 months
6	woman	Contractual	glands	13 years
7	woman	Official	surgery	27 years
8	woman	Contractual	General medical	15 years
9	woman	Contractual	surgery	6 months
10	Man	Official	orthopedic	10 years
11	Woman	plan based	heart	2 years
12	man	Contractual	General medical	3 years

Data analysis revealed 350 codes that were summarized into 36 codes classified into 10 subcategories and 4 categories as will be discussed below.

### Pioneering and priority care

Pioneering and prioritizing care were divided into two subcategories: “being a pioneer for care” and “prioritizing care”. Being a pioneer for care was related to rushing to attend the ward, rushing to start care, readiness for emergencies, and performance of routines. When dealing with their patients, nurses should assess them and identify their care needs. At the same time, by examining the patient kardex, nurses should extract the set of care that is necessary during the work shift, create a mental framework for themselves, and perform some care earlier than the due date in the care plan. Thus, they are always one step ahead of what they should do in this planned framework and are moving forward. They may also sometimes attend their shift earlier than the scheduled time, start the care earlier, and prepare themselves to face emergencies by anticipating and performing the ward’s routines to provide care as a model in providing anticipated care.

“*I often see some nurses who have to start their shift in the afternoon, attend the ward very early, i.e. at noon, and start checking the patient kardex and file to find out the procedures that shall be done by them*” (Man, , 8 months’ work experience).

According to the participants’ statements, care is prioritized by identifying the care, estimating the missed care, and determining the order or arrangement of the care. The participants reported that nurses should identify the total care that should be done from the very beginning of the shift. Then, based on their experience, their knowledge of the condition of the patients, and the prevailing conditions in the ward, they identify the care that is likely to be missed or delayed. Finally, they arrange and provide the total care needed by the patient through a mental plan. Hence, the nurses make a choice and prioritize some care and perform them earlier than the scheduled time:

“*Sometimes it happens that a patient needs to take several medicines in one hour or has a blood transfusion at the same time, and has to receive a few liters of serum. Thus, the patient has a busier schedule than other patients. If the nurse decides to provide the care according to the time detailed in the kardex, there is a high probability that the patient will not receive one or two drugs or that some nursing procedures will be missed for the patient. That’s why the nurse decides to prioritize some procedures and perform them ahead of time due to the patient’s condition. For instance, she gives the medicines earlier so that the blood transfusion can be also performed for the patient*” (Woman, , 12 years work experience).

### The range of anticipated care

Anticipated care involves a wide range of nursing procedures including “medication”, “serum therapy”, “clinical procedures” and “patient preparation for diagnostic procedures”. According to the nurses’ statements, the preparation and administration of the patient’s medicines shall be done at least one hour and at most two hours earlier than the prescribed time recorded in the patient’s kardex. Besides, most serums are prepared and administered as predicted: “*Many patients in the ward need to receive several liters of serum*, *take antibiotics*, *and also receive blood at the same time*. *Thus*, *a nurse should anticipate and perform some procedures earlier than the scheduled time so that she can perform all these tasks*. *That’s why most nurses start administering serums and drugs an hour earlier*” (Woman, , 13 months’ work experience).

Anticipated clinical procedures include blood sampling for tests, monitoring of vital signs, catheterization, oxygen therapy, suctioning, and intubation. Often, due to the overlap between the time of blood collection and the time of monitoring vital signs, most nurses perform this care as predicted at least one to two hours earlier than the scheduled time: “*I am working on the night shift*. *At 6 in the morning*, *I have to give medicines*, *control the patient’s signs*, *chart the procedures*, *take blood samples*, *and collect samples one or two hours earlier for most of the tests*” (Woman, , 13 years work experience).

Other clinical measures are carried out predictably depending on the working situation, the patient’s conditions, and the nurse’s clinical reasoning. Thus, given the inaccessibility of the doctor and the patient’s emergency condition, the nurses evaluate the patient’s condition based on their clinical reasoning and perform some measures such as inserting a catheter or administering oxygen, etc.: “*Sometimes there is a critically ill patient who is coded and needs CPR*, *but sometimes it happens that there is no time and the CPR team arrives late*, *and the nurses themselves predict the patient’s need for intubation and they will intubate the patient*” (Woman, , 12 years work experience).

The findings also showed that the nurses need to prepare the patient for diagnostic and therapeutic procedures such as spinal fluid sampling, bone marrow sampling, or taking the patient to the operating room for diagnostic or therapeutic surgery, while the patient is kept fasting. One of the participants stated: “*When we revive the results of the patient’s tests*, *we find that the patient has a low cell count*. *The patient’s symptoms*, *e*.*g*., *paleness and subcutaneous bleeding*, *and his/her medical records indicate that the patient will probably need bone marrow sampling tomorrow morning*. *So*, *we advise the patient not to eat anything until tomorrow morning to be ready for anticipated medical tests*” (Woman, , 12 years work experience).

### Antecedents of anticipated care

The findings showed that individual factors, organizational factors, and patient-related factors can contribute to anticipated care. According to the participants’ statements, individual factors related to nurses, including compassion, commitment to complete care, clinical reasoning, and time management are effective in creating anticipated nursing care. Thus, every nurse has a specific working framework for themselves, which is drawn implicitly as a model in the nurse’s mind and guides the way of providing care. Compassion and commitment to complete while focusing on managing time can lead nurses to perform anticipated care. Besides, nurses’ clinical reasoning in various encounters is a strong potential force that can contribute to providing anticipated care.

“*The thing that makes me perform anticipated care is my sense of sympathy for the patient and that I put myself in the patient’s shoes, and this makes me want to do something for him/her*” (Woman, , 2 years work experience).

Organizational factors including the workload of the ward, the working atmosphere, and nurses’ access to doctors can motivate nurses to perform anticipated care. The nurses in this study stated that under some circumstances such as the presence of several critically ill patients at the same time, the high workload of nurses, the overlap between the procedures in the ward, and the large number of patients compared to the nurses, nurses have to perform some procedures earlier than the scheduled time so they can provide care to all patients and do not miss or postpone any procedure: “*Sometimes when a nurse starts her shift*, *she will find that there are several critically ill patients or there are some patients that need to undergo multiple procedures*. *Thus*, *if the nurse does not take any action immediately*, *she cannot handle all procedures*. *That’s why she decides to perform some procedures earlier than the appointed time*” (Woman, , 27 years work experience).

The working atmosphere involves the degree of interaction and cooperation between colleagues, the routinized procedures in the ward, and colleagues’ mutual trust in each other. The way nurses support each other during the work shift is an important issue. Moreover, when a patient needs a doctor’s visit based on the assessment made by the nurse, but the doctor is not available for some reason, the nurse must take some action immediately based on her assessment of the patient’s condition. Thus, they anticipate and perform some care and procedures for the patient: “*We have some colleagues who are in charge of patients who don’t need much care and attention*. *They sit relaxed in a corner and do not help other nurses with critically ill patients who need to undergo many nursing procedures*. *So*, *we have to do some procedures earlier than the scheduled time*. *Some nurses do not do things well and tend to cut corners*. *Thus*, *we don’t ask for help from them and prefer to do the procedures on our own*” (Man, , 10 years work experience).

The findings also indicated that patient-related factors including patient acuity, patient expectations, old age, and having stable conditions also contributed to performing anticipated care. The acute conditions that caused the nurses to provide anticipated care were cardiac and respiratory arrest, pain, allergic reactions, and bleeding. In such circumstances, the passage of time is very important and the nurse should provide care as soon as possible. Furthermore, the unavailability of the doctor, patient expectations, and patient acuity lead to the choice of anticipated care: “*Sometimes we see a distressed patient who is coded*. *We page the CPR team*, *but it takes a long time for the anesthesia resident and the CPR team to attend the patient’s bedside*. *So*, *we have to predict some procedures and do them immediately due to the patient’s acute conditions and we cannot wait anymore*” Man, , 2 years work experience).

### Anticipated care consequences

The consequences of anticipated care include nurse-related consequences and patient-related consequences. The consequences experienced by the nurses after providing the anticipated nursing care were “feeling of guilt” and “peace of mind” and the consequences related to the patient were “satisfaction”, “improving the patient’s condition” and “getting hurt”. The participants in this study reported that they had experienced these two consequences following the provision of anticipatory care. Sometimes, by performing this type of care, which led to the improvement of the patient’s condition and the patient’s satisfaction with the nursing care, the nurses also felt a sense of inner satisfaction and peace of mind. In contrast, when the provision of anticipated care worsened the patient’s conditions or led to some complications, the nurses felt guilty for providing this type of care.

“*Sometimes a patient had a problem with urinating, and the nurses predicted that the patient needed a catheter, but catheterization caused damage to the patient’s urethra, and then they found out that he/she had a prostate problem and should not have used a catheter and thus they were feeling guilty for the harm made to the patient*” (Woman, , 6 months’work experience).

“*Sometimes a patient comes to you and you take blood from him and send it for testing anticipating that he needs*, *for example*, *CRP*, *but then the patient is visited and the doctor says that it was not necessary at all*. *So*, *you feel guilty for taking extra blood from the patient*” (Woman, 6 months’work experience).

## Discussion

This study examined the perception of nurses about anticipated nursing care. The findings showed that anticipated nursing care is perceived as “pioneer and priority care” and is characterized by “being a pioneer for care” and “prioritizing care”. In line with this finding, Bottega et al. (2020) also examined nurses’ perceptions and concluded that anticipated nursing care is care that is provided earlier than the expected time by nurses in the patient care plan, and the decision to provide this type of care is made by the nurse [18]. Thus, the nurse makes some arrangements to complete the procedures ahead of time. Accordingly, in contrast to the phenomenon of missed nursing care, there is another opposite phenomenon in which most of the interventions are anticipated [1] and the readiness of the nurse to manage unexpected cases and emergencies, because a reason for providing anticipated nursing care is to reduce the risk of omitting or postponing some care services [13, 16].

The findings also showed that anticipated nursing care involves a wide range of procedures including medication, serum therapy, clinical procedures, and preparing the patient for diagnostic-therapeutic procedures. Likewise, Bottega et al. (2020) reported that anticipated nursing care includes administering medicines, moving the patient, changing dressings, morning hygiene, controlling vital signs, taking blood samples, and performing some administration procedures [18]. Furthermore, Larnardelli et al. (2020) showed that anticipated care includes administering drugs, especially oral drugs, collecting samples for blood tests, changing dressings, changing angiocaths, preparing a bed for admitting a new patient and waking up the patient for primary care [24]. It seems that the differences in the types of anticipated nursing care reported in the literature can be attributed to the characteristics of the care bed, the duties of the members of the care team, and the management of the care needed by the patients. For instance, the participants in the present study reported that moving the patient and performing morning hygiene were usually performed by the patient’s caregivers or service personnel under the supervision of nurses, and the dressings were changed by medical students completing internship or residency courses in the hospital.

Other anticipated care services including monitoring vital signs and taking blood samples for testing were reported in the present study, similar to the two studies reviewed above. Kalish et al. examined missed nursing care and showed that vital signs control and blood sugar control are among the missed care [31]. Borzoui et al. (2016) reported that the most frequent missed nursing care in the adult ICU was the control of vital signs [32]. In addition, Rezaei et al. (2019) stated that one of the most frequently missed care is blood sugar control [33]. As such, it seems that nurses tend to identify the care with a greater chance of being forgotten and predictably perform it.

Preparing the patient for performing diagnostic-therapeutic procedures was another anticipated care reported by the nurses in the present study. The nurses reported that they often performed required procedures by keeping the patient fasting, preparing the necessary items, and completing the relevant documentation as predicted. In line with this finding, Bottega et al. (2020) also reported clinical records and notes, especially for patients who were in stable condition, and providing the equipment needed by the patients before the equipment in the ward was exhausted as anticipated care performed by nurses [18].

Following the participants’ statements, individual factors, organizational factors, and patient-related factors can contribute to anticipated care. The factors are intertwined with each other, and together they serve as a driving force for decision-making by the nurse to provide anticipated care. Individual factors included compassion, commitment to completing care, clinical reasoning, and time management. The participants stated that compassion is an important factor in the provision of anticipated care and that patients expect to receive compassionate care. According to professional standards, nurses are also expected to communicate with patients through compassionate behaviors to respond to their pain and suffering [34] and when the nurses put themselves in the patient’s place and look at the issues from the patient’s perspective, they understand the patient and provide compassionate care and assistance for the patient [35]. Indeed, nurses’ knowledge of professional values and how they affect nurses’ behaviors is an important factor in nursing care [36]. Thus, nurses adhere to these values to make decisions to provide care and face ethical issues [37]. Moreover, adherence to professional values can prevent the occurrence of missed care [38] and contribute to offering anticipated care [18], encouraging nurses to try to improve their professional performance [39].

The participants in this study also reported that commitment to completing care is another personal characteristic leading nurses to perform anticipated care. In line with this finding, Bottega et al. (2020) also stated that there are unwritten duties among nurses, one of which is to leave the patient and the ward regularly and completely for the next shift colleague, and all care must be completed before the end of the shift to prevent an unfavorable work shift for other colleagues and not to impose additional duties on them. Thus, this type of commitment prevents missed care and helps other nurses perform all required procedures before the end of their shift and is considered a sign of respect for the nurses working on the next shift [18].

Nurses’ clinical reasoning is another motivating factor for performing anticipatory nursing care as reported by the participants in this study. Accordingly, an increase in nurses’ work experience has an effective role in clinical reasoning. Thus, nurses with clinical reasoning assess the patient’s condition as soon as they encounter the patient and combine the data obtained with their knowledge, and through clinical reasoning, quickly decide to implement care. Indeed, clinical reasoning is a cognitive process used for clinical judgment. In this process, the patient’s medical history is reviewed, a physical assessment is performed, and the results are interpreted to design a care plan [40]. Thus, nurses obtain information to solve the patient’s problem and combine this information with their knowledge to guide decision-making for patient care [41]. Moreover, nurses who have clinical reasoning can provide better services for their patients and their decisions are safer and of higher quality [42].

Finally, time management is another individual factor affecting anticipated nursing care. Nurses often have many responsibilities, including coordinating requested consultations and reporting to doctors and nutrition and physical therapy consultants. They have to spend a lot of time admitting and discharging patients and performing non-nursing administrative procedures, so they often run out of time [43] and due to time constraints, they prioritize care so that they can perform care with higher priority in limited time and with limited resources [44]. In addition, unexpected, additional, and repetitive tasks such as answering phones, handling requests from visitors, and administering drugs with an urgent prescription order can affect the nurse’s ability during the limited time she has, leading to missed care [45]. As a result, when planning care, nurses specify a set of tasks that are more time-consuming and do simpler tasks first and complete them until there is enough time to perform other nursing interventions. Furthermore, they first perform the interventions with anticipated completion time and they allocate the rest of their time for care that cannot be estimated [24].

The second antecedent of anticipated nursing care was the organizational factors, including the workload of the ward, the working atmosphere, and nurses’ access to doctors. The shortage of medical staff and the high workload of the ward interact and together with individual factors make nurses provide anticipated care. A review of the literature indicates that nurses often prioritize and perform care based on system expectations and physician preferences, resulting in the prioritization of primary and emergency care such as administering medications, intravenous treatments, or activities observable for managers [46, 47]. Furthermore, when the number of patients is high compared to nurses, each additional patient leads to an increase in the nurse’s workload, and the workload has a significant effect on the occurrence of missed care. Thus, an increase in the number of patients in proportion to nurses increases the probability of missed care by 70%. Thus, to prevent the risk of missed care and to have the best performance, nurses tend to engage in providing anticipated nursing care [18].

In addition, the working atmosphere plays a major role in choosing the anticipated care. The participants reported that the working atmosphere involves the degree of interaction and cooperation between colleagues, the routinized procedures in the ward, and colleagues’ mutual trust in each other. The communication between the members of the care team can contribute to the continuity of care and help in reducing the frequency of missed care [48], and in the care setting with a high level of missed care, there is no adaptive behavior among the staff. Adaptive behavior means that instead of focusing on “my patient”, the nurse focuses on “our patients”. Thus, based on the number of patients, the acuity of the disease, and fluctuations in the number of medical staff, nurses with such a perspective tend to adopt collective orientation and situational awareness, and with continuous evaluation, they try to identify the work requirements of the members of the care team and provide care to help each other [9]. In contrast, when there is no collective spirit, there will be no communication and cooperation among nurses, paving the way for the occurrence of missed care [24].

As such, nurses are more likely to provide anticipated care as the main strategy to prevent the occurrence of missed care. Moreover, the lack of trust in the nursing team along with the professional accountability and work habits and routines in the ward that were formed based on expectations and beliefs and were accepted unquestionably (the reutilization of anticipated care procedures as the old pattern common in the ward) can increase the desire to perform anticipated activities [24].

The nurse’s access to the doctor is another determining factor for providing anticipated care. The participants stated that the doctor’s failure to attend to the patient’s bedside on time to visit him/her and provide treatment orders, the patient’s expectations from medical staff to meet the patient’s needs, and the direct contact between the nurse and the patient, and the nurse’s compassion for meeting the patient’s needs can contribute to starting anticipated nursing care. Indeed, the collaborations between doctors and nurses are effective in meeting the patient’s goals [49] and such collaborations can be affected by the shortage of doctors. Thus, the larger number of patients compared to the number of doctors in hospitals increases the workload of doctors and thus they feel that they should be everywhere, but they often cannot respond to all the calls from nurses to handle the patients’ needs in the wards [50].

Furthermore, patient-related factors including the acuteness of the patient’s condition, the patient’s expectations, old age, and having stable conditions can also put nurses in a decision-making position to provide anticipated care because patients expect to be quickly admitted and their pain and suffering are relieved. All patients are concerned about meeting their needs and they demand quick care [51]. In addition, relieving the patient’s pain is considered a fundamental right of the patient and a part of the care priorities and moral and legal right of the patient [52] and to fulfill the right of the patient, the nurse should provide her care according to the patient’s needs within a relationship established based on trust [53]. In addition, patients who are selected to receive the anticipated care are mostly older patients and patients with stable conditions [24] as older patients are considered a sensitive group and need more respect and attention [54] and healthcare workers should focus on what the elderly want [55]. Moreover, patients with a stable condition are clinically stable. Therefore, nurses perform some anticipated procedures for these patients including completing their medical files and records [18].

However, it is worth mentioning that studies that have addressed missed nursing care which is in sharp contrast to anticipated nursing care have reported similar organizational factors such as “medical staff under pressure” [43], the number of medical staff compared to the number of patients [46, 47], lack of time [56], weak teamwork [48], team breakdown, and ineffective communication and cooperation between nurses and doctors [24]. Interestingly, these factors affect both missed and anticipated nursing care. Thus, it can be argued that to prevent the occurrence of missed care, nurses tend to engage in providing anticipated care. As such, nursing managers should take into account these factors and manage situations influenced by these factors.

The findings from the present study also showed that the consequences of anticipated care include “patient-related consequences” and “nurse-related consequences”. The consequences experienced by the nurses after providing the anticipated nursing care were “feeling of guilt” and “peace of mind” and the consequences related to the patient were “satisfaction”, “improving the patient’s condition” and “getting hurt”. In line with these findings, Bottega et al. (2020) reported that the consequences of anticipated care included patient and nurse-related consequences. The consequences related to the patients include “reducing the quality of nursing care, reducing the risk of missed care, and increasing patient satisfaction” and the consequences related to the nurse include “increasing the capacity of facing unpredictable events” and “increasing cohesion and sense of respect between colleagues” [18].

Moreover, Lanardelli et al. (2020) reported that the consequences of anticipated care include “patient-related consequences” and “nurse-related consequences”. One of the nurse-related consequences was avoiding the imposition of procedures on the colleagues in the next shift, which created a sense of peace, and satisfaction, increased cooperation and solidarity between colleagues, and patient-related consequences were the reduced risk of missed care and increased patient satisfaction [24].

### Study limitations

This study had some limitations, such as the fact that it only examined the perceptions of nurses of different medical and surgical departments of a university-affiliated hospital, so when studying the findings, should pay attention to this issue, because examining the perceptions of nurses in other departments such as intensive care unit, pediatric, newborn and emergency departments and other hospitals, according to organizational characteristics and methods of providing nursing care may appear different results so the researchers of this study were faculty members of the university in nursing field and therefore their perceptions and assumptions about the nursing care may have biased their interpretation of the data. The data were analyzed separately by each researcher and dialogue led to consensus on thematic categories.

## Conclusion

The findings from the present study indicated that the nurses perceived the anticipated care as pioneering and priority care. Anticipated care is performed earlier than the scheduled time and involves a wide range of nursing procedures including medication, serum therapy, performing clinical procedures, and preparing the patient for diagnostic-therapeutic procedures. Moreover, some organizational factors such as the workload of the ward, the workplace atmosphere, the access of the nurse to the doctor, and individual factors including compassion, commitment to completing care, time management, and clinical reasoning can pave the way for providing anticipated nursing care.

The findings of the study can have some implications for nursing managers for improving the quality of nursing care by taking some actions such as planning for recruiting nursing staff and allocating them based on the workload in different departments. Moreover, hospital managers and planners can focus on developing continuing educational programs with a focus on teamwork skills, communication, professionalism, and changing the routine procedures of providing nursing care. Moreover, the insights from the present study can contribute to developing comprehensive and practical assessment tools in clinical audits to pave the way for fulfilling the ultimate goal of the healthcare system, which is to improve the quality of nursing care.

Iit is suggested to conduct further studies with the aim of explaining perception of patients or intensive care unit nurses in relation to anticipated nursing care so development and psychometric assessment an instrument for investigating anticipated nursing care can provide the basis for conducting quantitative studies in this field.

## Supporting information

S1 File Contains supplementary table categories and subcategories. (DOCX)
