# Supplementary material for: Nurses’ perception of anticipated nursing care: A qualitative research
Source: PLoS One. 2025 Feb 12;20(2):e0308257. doi: 10.1371/journal.pone.0308257 (PMC11819522; doi:10.1371/journal.pone.0308257)
Supplement: S1 File — (DOCX) [file pone.0308257.s001.docx]

| **Themes** | **categories** | **subcategories** |
| --- | --- | --- |
| Pioneering and priority care | Being a pioneer for care | to rushing to attend the ward |
|  |  | rushing to start care |
|  |  | readiness for emergencies |
|  |  | performance of routines |
|  | prioritizing care | identifying the care |
|  |  | estimating the missed care |
|  |  | arrangement of the care |
| The range of anticipated care | Medication and  serum therapy | prepare drugs |
|  |  | administration of medicines |
|  |  | prepare serums |
|  |  | administration of serums |
|  | clinical procedures | blood sampling |
|  |  | monitoring of vital signs |
|  |  | catheterization |
|  |  | oxygen therapy |
|  |  | suctioning |
|  |  | intubation |
|  | Prepare patient for diagnostic procedures | kept the patient fasting |
|  |  | completed the patient's medical file |
| Antecedents of anticipated care | individual factors | compassion |
|  |  | commitment to complete care |
|  |  | clinical reasoning |
|  |  | time management |
|  | organizational factors | workload of the ward |
|  |  | the working atmosphere |
|  |  | nurses’ access to doctors |
|  | patient-related factors | Patient acuity |
|  |  | patient’s expectations |
|  |  | old age |
|  |  | having stable conditions |
| Anticipated care consequences | nurse-related consequences | feeling of guilt |
|  |  | peace of mind |
|  | patient-related consequences | satisfaction |
|  |  | improving the patient’s condition |
|  |  | getting hurt |
